# Modelling subject-specific childhood growth using linear mixed-effect models with cubic regression splines

**DOI:** 10.1186/s12982-015-0038-3

**Published:** 2016-01-07

**Authors:** Laura M. Grajeda, Andrada Ivanescu, Mayuko Saito, Ciprian Crainiceanu, Devan Jaganath, Robert H. Gilman, Jean E. Crabtree, Dermott Kelleher, Lilia Cabrera, Vitaliano Cama, William Checkley

**Affiliations:** Program in Global Disease Control and Epidemiology, Department of International Health, Bloomberg School of Public Health, Johns Hopkins University, Baltimore, USA; Department of Mathematical Sciences, Montclair State University, Montclair, NJ USA; Asociación Benéfica PRISMA, Lima, Peru; Departamento de Microbiología, Universidad Peruana Cayetano Heredia, Lima, Peru; Department of Biostatistics, Bloomberg School of Public Health, Johns Hopkins University, Baltimore, USA; Leeds Institute of Molecular Medicine, St James’s University Hospital, Leeds, UK; School of Medicine, Trinity College Dublin and Dublin Molecular Medicine Centre, Dublin, Ireland; Division of Parasitic Disease, Centers for Disease Control, Atlanta, USA; Division of Pulmonary and Critical Care, Department of Medicine, Johns Hopkins University, 1800 Orleans Ave Suite 9121, Baltimore, MD USA

**Keywords:** Longitudinal studies, Body Height, Child development, Growth, Linear Models

## Abstract

**Background:**

Childhood growth is a cornerstone of pediatric research. Statistical models need to consider individual trajectories to adequately describe growth outcomes. Specifically, well-defined longitudinal models are essential to characterize both population and subject-specific growth. Linear mixed-effect models with cubic regression splines can account for the nonlinearity of growth curves and provide reasonable estimators of population and subject-specific growth, velocity and acceleration.

**Methods:**

We provide a stepwise approach that builds from simple to complex models, and account for the intrinsic complexity of the data. We start with standard cubic splines regression models and build up to a model that includes subject-specific random intercepts and slopes and residual autocorrelation. We then compared cubic regression splines vis-à-vis linear piecewise splines, and with varying number of knots and positions. Statistical code is provided to ensure reproducibility and improve dissemination of methods. Models are applied to longitudinal height measurements in a cohort of 215 Peruvian children followed from birth until their fourth year of life.

**Results:**

Unexplained variability, as measured by the variance of the regression model, was reduced from 7.34 when using ordinary least squares to 0.81 (p < 0.001) when using a linear mixed-effect models with random slopes and a first order continuous autoregressive error term. There was substantial heterogeneity in both the intercept (p < 0.001) and slopes (p < 0.001) of the individual growth trajectories. We also identified important serial correlation within the structure of the data (ρ = 0.66; 95 % CI 0.64 to 0.68; p < 0.001), which we modeled with a first order continuous autoregressive error term as evidenced by the variogram of the residuals and by a lack of association among residuals. The final model provides a parametric linear regression equation for both estimation and prediction of population- and individual-level growth in height. We show that cubic regression splines are superior to linear regression splines for the case of a small number of knots in both estimation and prediction with the full linear mixed effect model (AIC 19,352 vs. 19,598, respectively). While the regression parameters are more complex to interpret in the former, we argue that inference for any problem depends more on the estimated curve or differences in curves rather than the coefficients. Moreover, use of cubic regression splines provides biological meaningful growth velocity and acceleration curves despite increased complexity in coefficient interpretation.

**Conclusions:**

Through this stepwise approach, we provide a set of tools to model longitudinal childhood data for non-statisticians using linear mixed-effect models.

**Electronic supplementary material:**

The online version of this article (doi:10.1186/s12982-015-0038-3) contains supplementary material, which is available to authorized users.

## Background

Childhood growth is a cornerstone of pediatric research and many centuries of work have been undertaken to understand and model how children grow [1–5]. In studies of childhood growth, anthropometric data are often collected at multiple time points to describe growth in a population, evaluate the role of exposures on growth, investigate the effects of an intervention, and assess individual growth in clinical practice [6–11]. Height is commonly monitored longitudinally as a marker of chronic malnutrition; however, estimation and prediction of subject-specific height curves with age, as in the study we consider here, can present several methodological challenges to researchers.

Cross-sectional studies are an attractive option for surveillance because of their feasibility and cost-effectiveness in populations, but this approach for growth monitoring has several inherent limitations. For example, they can be confounded by secular trends, such as selective mortality that leads to perceived improved growth at older ages due to the better health of the survivor population [12]. Additionally, cross-sectional growth data may display large skewness and kurtosis and may exhibit substantial heteroskesdasticity, and marginal analyses to describe population trajectories require transformations to normality, weighting or both to achieve an adequate fit [13]. While longitudinal data may also suffer from the same problems, linear mixed effects models naturally take skewness, kurtosis, and even heteroskedasticity into account, making transformations not necessary. The utility of transformation techniques remains controversial. Indeed, while transformations may lead to a better fit of Gaussian models, they require a priori knowledge of the data structure. The flexible Box-Cox transformation family of distributions [14, 15] can be used, but it may fail when data are clustered. Moreover, interpretation of transformed data is problematic, and producing predictions at the subject and population level is not straightforward.

Longitudinal studies provide a more realistic view of child growth, in which a cohort of children are monitored over time and repeated measurements of height are collected. Longitudinal studies provide information about individual growth patterns, and allow the estimation and analysis of growth velocity and acceleration at either the individual or population level. However, longitudinal growth data has complex characteristics that need to be accounted for including: within-subject clustering of growth observations, heterogeneity of individual baseline and dynamic growth characteristics, and autocorrelation within individuals.

Linear mixed-effect models combine the components of fixed effects, random effects, and repeated measurements in a single unified approach [16, 17]. Analysis of longitudinal data using mixed effects models does not require the same assumptions as a cross-sectional study and may not require transformations. The use of linear mixed-effect models, while widely described in the statistical literature, has been only slowly adopted by applied researchers. This was due in part to the limited availability of user-friendly software tools; this is now rapidly changing with many commercial and open source software providing fitting capabilities for increasingly complex mixed effects models [18]. To address this problem, ensure reproducibility of methods, and provide a wider dissemination, we provide examples of statistical code in an open-source platform for fitting models described in this paper. Another reason for the slow adoption of linear mixed-effect models is their complexity relative to standard regression models and the modeling structures necessary to capture non-linear trajectories. These elements require a higher level of statistical and computational expertise [19]. Utilizing longitudinal length/height data from a child cohort in Peru, we describe a natural and intuitive stepwise approach to the development of a linear mixed-effect model with cubic splines for the analysis of longitudinal childhood growth in height. We then derive individual height velocity and acceleration curves from the longitudinal model based on height. Our objective is to describe the use of these methods to analyze height and height velocity data in a way that can be easily used by an applied researcher. While we have a preference to use the raw measurements of growth, methods introduced here extend directly to Z-scores relative to the WHO standard.

## Methods

### Study setting

The study was conducted in Las Pampas de San Juan Miraflores and Nuevo Paraíso, both peri-urban shanty towns with high population density located on the southern edge of Lima city in Peru. The shanty towns had approximately 40,000 residents of whom 25 percent are under 5 years of age. These communities are described in detail elsewhere [10, 11].

### Study participants

A simple census was conducted to find pregnant women and children less than 3 months of age. Eligible newborns and pregnant women were randomly selected from the census and invited to participate in the study. Only one newborn was recruited per household. Written informed consent was required from parents or guardians before enrollment. Exclusion criteria for the study were: severe disease that required hospitalization, severe or chronic conditions, child of a multiple pregnancy, birth weight less than 1500 g and plans to move to another community within the study time. Our analysis excluded data of children whose follow-up ended before they reached one year of age, those who were followed up for less than 60 days during the first 6 months of life, or followed up for less than 120 days during the first year of life.

### Study design

We conducted a birth cohort study between May 2007 and February 2011. Child growth data presented in this paper is a part of a larger longitudinal study conducted in Brazil and Peru. The objective of the cohort study was to assess if infection with *Helicobacter pylori* increases the risk of diarrhea and in turn adversely affects growth in children less than 2 years of age [20]. For the purposes of illustration, this study utilizes longitudinal height data from Peru only.

### Outcomes

We measured anthropometrics weekly until the child was 3 months of age, every 2 weeks between three and 11 months of age, and once monthly thereafter for the remainder of follow-up. Both height and recumbent length (supine length) was measured using a wooden length platform and sliding head-footboard (Shorr stadiometers, Shorr Productions, Olney, Maryland). We followed calibration procedures as per manufacturer instructions. Height or length were measured to the nearest 0.1 cm. Anthropometric standardization was conducted at the beginning of the study, and once yearly thereafter. Outcomes of this study were height in centimeters and height velocity in centimeters per month. Child length at birth was obtained from the perinatal care booklet given to mothers from the health care providers. Children less than 2 years of age were measured in a horizontal position (recumbent length), whereas children aged 2 years and older were measured in a vertical position (height). Length (or height) was measured using a wooden length platform and sliding footboard (or headboard) to the nearest 0.1 cm.

Height velocity was calculated in two ways. First, we calculated empirical height velocity by subtracting previous height from current height and dividing by the time gap between both measurements:

$$\varDelta height(t_{ij} ) = \frac{{height\left( {t_{ij} } \right) - height(t_{ij - 1} )}}{{t_{ij} - t_{ij - 1} }}$$

Second, we computed an estimated height velocity using the first time derivative of the longitudinal height models using standard methods (Table 1).

**Table 1:** Representation of three common forms of regression splines and calculation of first derivatives

	Regression equation	First derivative
Truncated polynomial splines (order p)	$f_{TPS} ( x ) = \beta_{0} + \beta_{1} x + \cdots + \beta_{p} x^{p} + \sum_{k = 0}^{K - 1} \gamma_{k} ( {x - \xi_{k} })_{ + }^{p}$ where $$( {x - \xi } )_{ + } = \left\{ \begin{array}{*{20}ll} 0 &\quad if \,\, x \le \xi \\ x - \xi &\quad if \,\,x > \xi \\ \end{array} \right.$$	$f_{TPS}^{'} \left( x \right) = \beta_{1} + 2\beta_{2} x^{2} + \cdots + p\beta_{p} x^{p - 1} + \mathop \sum \nolimits_{k = 0}^{K - 1} p\gamma_{k} \left( {x - \xi_{k} } \right)_{ + }^{p - 1}$
B-splines (order p)	$f_{B} ( x ) = \sum_{k = 0}^{K - p - 2} \alpha_{k} B_{k,p} ( x )$ where $$B_{k,0} ( t ) = \left\{ \begin{array}{*{20}ll} 1 &\quad if \,x_{k} \le x < x_{k + 1} \\ 0 & \quad if \,otherwise \end{array} \right.$$ and higher order splines bases are obtained by the recursion $B_{k,p} \left( x \right) = \frac{{x - x_{k} }}{{x_{k + p} - x_{k} }}B_{k,p - 1} \left( x \right) + \frac{{x_{k + p + 1} - x}}{{x_{k + p + 1} - x_{k + 1} }}B_{k + 1,p + 1} \left( x \right)$	$b_{k,0}^{'} ( x ) = 0$ $b_{k,p}^{'} ( x ) = \frac{1}{{x_{k + p} - x_{k} }}B_{k,p - 1} ( x ) + \frac{{x - x_{k} }}{{x_{k + p} - x_{k} }}B_{k,p - 1}^{'} \left( x \right) - \frac{1}{{x_{k + p + 1} - x_{k + 1} }}B_{k + 1,p - 1} \left( x \right) + \frac{{x_{k + p + 1} - x}}{{x_{k + p + 1} - x_{k + 1} }}B_{k + 1,p - 1}^{'} \left( x \right)$
Natural cubic splines (order 3)	$$\begin{array}{*{20}ll} f_{{ns}} \left( x \right) &=\, \beta _{0} + \beta _{1} x + \sum_{{k = 0}}^{{K - 1}} {\gamma _{k} } \left( {x - \xi _{k} } \right)_{ + }^{3} \\& \quad \sum_{{k = 0}}^{{K - 1}} {\gamma _{k} } \xi _{k} = \sum_{{k = 0}}^{{K - 1}} {\gamma _{k} } = 0 \end{array}$$	$$\begin{array}{*{20}ll} f_{ns}^{'} \left( x \right) & =\, \beta_{1} + \mathop \sum _{k = 0}^{K - 1} 2\gamma_{k} \left( {x - \xi_{k} } \right)_{+}^{2} \\ & \quad \sum _{k = 0}^{K - 1} \gamma_{k} \xi_{k} = \mathop \sum _{k = 0}^{K - 1} \gamma_{k} = 0 \\ \end{array}$$

### Biostatistical methods

The primary aim of our analysis was to model height and height velocity. We included the following predictors in our models: age in months, an indicator variable for greater than 24 months to account for unit differences in length vs. height measurement methods, and sex. To model the non-linear relationship between age and height over time, we used smooth, flexible functions known as cubic regression splines. While there are several forms of regression splines that can be used to model non-linear relationships between a predictor (i.e., age) and an outcome (i.e., height), we chose to use cubic regression splines because they are simple to construct and understand [21]. We purposely varied the number and positions of the interval knots in several of our examples to demonstrate that our models are not affected by these changes. As mentioned, derivation of different types of splines to calculate height velocity is straightforward (Table 1). Since other investigators [6] have proposed the use of linear splines to model child growth because of their ease of use, in this paper, we compared adequacy of both estimation and prediction between cubic and linear regression splines with variations in the number and positions of the knots. We compared cubic and linear regression spline models using an in-sample (i.e., estimation) mean square error (MSE) and out-of-sample (i.e., prediction) MSE using standard methods. For out-of-sample prediction, we used 80 % of the data for training and 20 % of the data for validation. For each individual growth curve, we randomly sampled 80 % of the data values and used them to construct the model fit. The validation data consisting of 20 % of the data was then used to generate predicted height values. The predicted height and observed height were used to compute subject-specific prediction MSEs.

As described in detail below, the following statistical methods were used in the model: a cubic regression spline with knots at 3, 6, 12, 18, 24 and 40 months, random intercepts and slopes to capture the heterogeneity in growth curves, and a first-order continuous autoregressive error [CAR(1)] to capture residual serial correlation that arises from repeated measurements of the same child [22–24]. At each step of model building, we conducted exploratory analysis in which we visualized standardized residuals with age, a sample variogram of the residuals [25], and pairwise scatterplots of residuals at different time points to assess goodness-of-fit. We limited the number of internal knots for our statistical models between three and six because this number is likely sufficient to model the inflexion of linear/height growth curves in children under 5 years of age. Moreover, we also tested slight variations in the numbers and positioning of knots to determine if the model fit was affected. We conducted our statistical analyses in STATA 11 (StataCorp LP, College Station, TX, USA) and R (http://www.r-project.org). Examples of statistical code in R are provided to ensure reproducibility and improve dissemination of methods (Additional file 1).

### Research ethics

This study was approved by the internal review boards of A.B. PRISMA (Lima, Peru), Johns Hopkins University (Baltimore, USA), and the European Union Ethics Committee.

## Results

### Baseline characteristics

We included a total of 215 children in this analysis. The initial cohort started with 304 children: however, 11 did not have available anthropometric data and 78 were lost to follow up before 1 year of age. Average follow-up was 34 months (range 12–45 months). Average number of observations per children was 50, giving a total of 10,838 observations. Of 215 children, 109 were girls (51.0 %).

### Initial observations

**Fig. 1:**
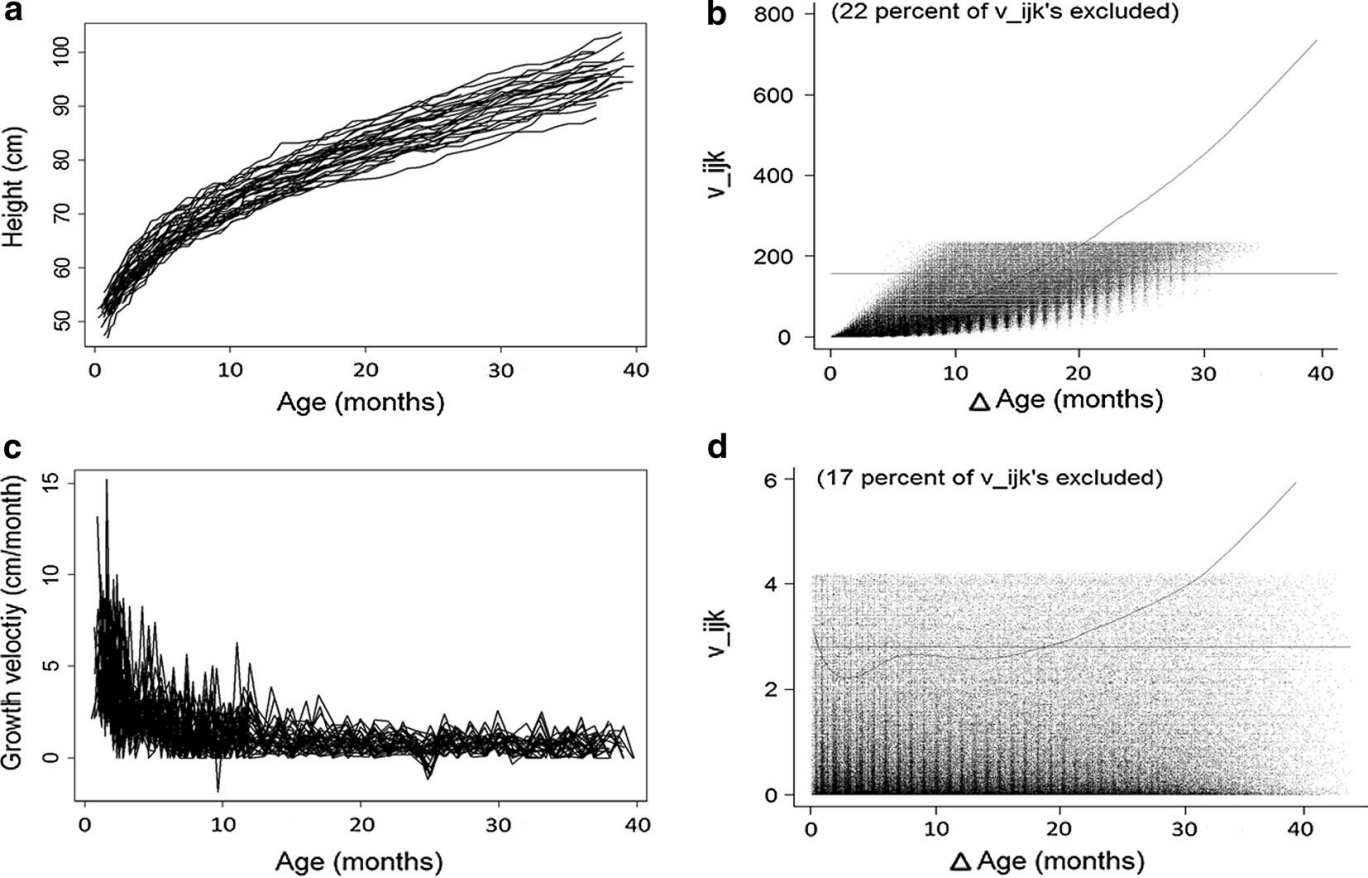
*Panels* **a**, **c** shows a spaghetti plot of the height and height velocity raw data from 50 participants respectively. Panels **b**, **d** show the variograms corresponding with each data set, where values in the x-axis represent the distance in time between two measurements and values in the y-axis (v_ijk_) represent the *square* distance between those two observations [29]

We conducted exploratory data analysis as the first step to gain insight into the structure and nature of the data. In exploratory analysis for longitudinal data, we obtained an understanding of the non-linear relationship of height with age but also the heterogeneity in growth curves for the study population (Fig. 1). Using a spaghetti plot of empirical height velocity, we found that it declined with age and the change was steepest at the youngest ages. In addition, in longitudinal growth data with repeated height measures, it is important to evaluate the serial autocorrelation, or the relationship between these measures. The variogram is a useful tool, as it evaluates autocorrelation by comparing the half square difference between each pair of heights within an individual to the time gap associated with each pair of heights. A nonparametric smooth fit is added to the variogram to describe the pattern. The exponential nonparametric smooth fit indicates that data exhibit both strong serial autocorrelation and heterogeneity (Fig. 1).

### Building a longitudinal growth model

Our exploratory data analysis highlighted several challenges that need to be addressed in modeling growth data. Specifically, we had to account for the shape of the curve, the clustering of values, the heterogeneity among individuals, and the individual effects of serial correlation. To emphasize the role of each of these factors, we used combinations of random effects and serial correlation components in a linear model (Table 2). The steps to build the final model are described below.

**Table 2:** Linear models used in our analyses

Model	Regression equations
Ordinary least squares	$$\begin{array}{*{20}ll} Height_{ij} &=\, \beta_{0} + \beta_{1} t_{ij} + \beta_{2} t_{ij}^{2} + \beta_{3} t_{ij}^{3} + \beta_{4} \left( {t_{ij} - 3} \right)_{ + }^{3} + \beta_{5} \left( {t_{ij} - 6} \right)_{ + }^{3} + \beta_{6} \left( {t_{ij} - 12} \right)_{ + }^{3} + \beta_{7} \left( {t_{ij} - 18} \right)_{ + }^{3}\\&\quad + \beta_{8} \left( {t_{ij} - 24} \right)_{ + }^{3} + \beta_{9} \left( {t_{ij} - 40} \right)_{ + }^{3} + \beta_{10} I\left( {t_{ij} > 24} \right) + \beta_{11} I\left( {male} \right) + \varepsilon_{ij} \end{array}$$ $\varepsilon_{ij} \sim N\left( {0, \sigma^{2} } \right)$
Linear mixed-effect model with random intercept and random slope.	$$\begin{array}{*{20}ll} Height_{ij}&=\, \beta_{0} + b_{0i} + \beta_{1} t_{ij} + b_{1i} t_{ij} + \beta_{2} t_{ij}^{2} + \beta_{3} t_{ij}^{3} + \beta_{4} \left( {t_{ij} - 3} \right)_{ + }^{3} + \beta_{5} \left( {t_{ij} - 6} \right)_{ + }^{3} + \beta_{6} \left( {t_{ij} - 12} \right)_{ + }^{3} \\ &\quad + \beta_{7} \left( {t_{ij} - 18} \right)_{ + }^{3} + \beta_{8} \left( {t_{ij} - 24} \right)_{ + }^{3} + \beta_{9} \left( {t_{ij} - 40} \right)_{ + }^{3} + \beta_{10} I\left( {t_{ij} > 24} \right) + \beta_{11} I\left( {male} \right) + \varepsilon_{ij} \end{array}$$ $$\left( {\begin{array}{*{20}c} {b_{0} } \\ {b_{1} } \\ \end{array} } \right)\sim {\text{MVN}}\left( {\left[ {\begin{array}{*{20}c} 0 \\ 0 \\ \end{array} } \right],\left[ {\begin{array}{*{20}c} {g_{11} } & {g_{21} } \\ {g_{12} } & {g_{22} } \\ \end{array} } \right]} \right)$$ $\varepsilon_{ij} \sim N\left( {0, \sigma^{2} } \right)$
Linear mixed-effect model with random intercept and random slope and first order continuous autoregression (CAR(1))	$$\begin{array}{*{20}ll} Height_{ij} &=\, \beta_{0} + b_{0i} + \beta_{1} t_{ij} + b_{1i} t_{ij} + \beta_{2} t_{ij}^{2} + \beta_{3} t_{ij}^{3} + \beta_{4} \left( {t_{ij} - 3} \right)_{ + }^{3} + \beta_{5} \left( {t_{ij} - 6} \right)_{ + }^{3} + \beta_{6} \left( {t_{ij} - 12} \right)_{ + }^{3}\\ &\quad + \beta_{7} \left( {t_{ij} - 18} \right)_{ + }^{3} + \beta_{8} \left( {t_{ij} - 24} \right)_{ + }^{3} + \beta_{9} \left( {t_{ij} - 40} \right)_{ + }^{3} + \beta_{10} I\left( {t_{ij} > 24} \right) + \beta_{11} I\left( {male} \right) + \varepsilon_{ij} \end{array}$$ $$\left( {\begin{array}{*{20}c} {b_{0} } \\ {b_{1} } \\ \end{array} } \right)\sim {\text{MVN}}\left( {\left[ {\begin{array}{*{20}c} 0 \\ 0 \\ \end{array} } \right],\left[ {\begin{array}{*{20}c} {g_{11} } & {g_{21} } \\ {g_{12} } & {g_{22} } \\ \end{array} } \right]} \right)$$ $$\varepsilon_{ij} \sim N\left( {\left[ {\begin{array}{*{20}c} 0 \\ \vdots \\ 0 \\ \end{array} } \right], \sigma^{2} \left[ {\begin{array}{*{20}c} 1 & \ldots & {\rho^{{\left| {t_{i1} - t_{{im_{i} }} } \right|}} } \\ \vdots & \ddots & \vdots \\ {\rho^{{\left| {t_{{im_{i} }} - t_{i1} } \right|}} } & \ldots & 1 \\ \end{array} } \right]} \right)$$

### Ordinary least squares model

Ordinary least squares (OLS) estimates growth parameters by minimizing the sum of the squared vertical distances between the observed and predicted responses. It provides efficient and valid predictions with the following assumptions: height can be explained by a linear combination of predictors, values of height are determined independently of each other, height has a normal distribution at any particular age, and height has equal variances at any particular age.

**Fig. 2:**
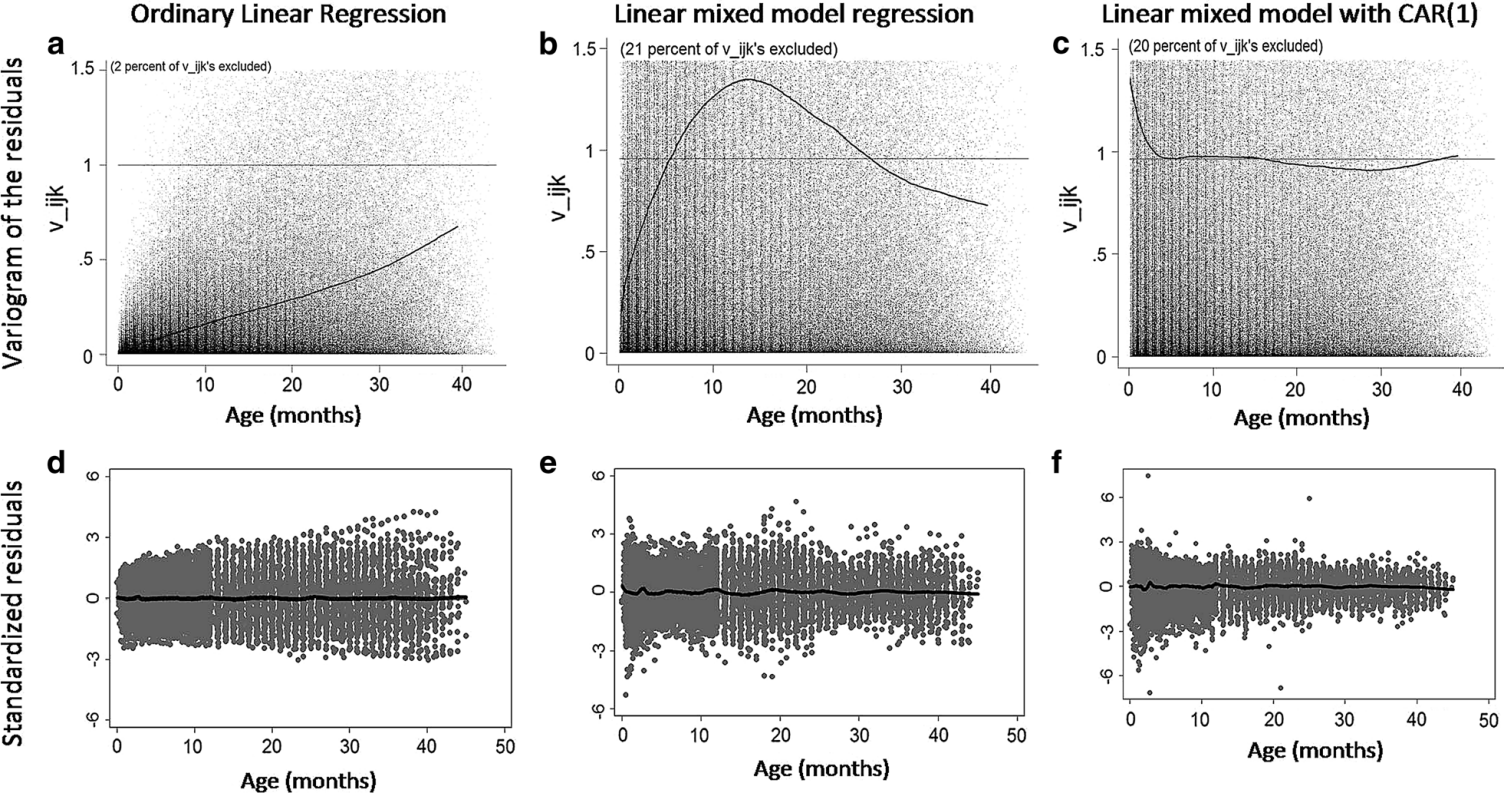
*Panels* **a**, **d** shows the variogram and standardized residuals for the fit using OLS. *Panels* **b, e** shows for mixed model regression. *Panels* **c**, **f** shows for linear mixed model with CAR(1). Notice how the fit improves with each new addition. In* Panels* **a**–**c**, values in the x-axis represent the distance in time between two measurements and values in the y-axis (v_ijk_) represent the square distance between those two observations [29]

Unfortunately, growth data violates all of these assumptions. First, growth does not follow a linear pattern with age. This can be addressed through the use cubic regression spline to model the curvature with age. However, there are other concerns that cannot be as easily adjusted by OLS. Several height measurements were taken from each child; however, these serial measurements of height for the same child are not independent of each other. This is confirmed by a variogram of the residuals from the OLS (Fig. 2, panel a). Next, the height data did not follow a normal distribution as per the Shapiro–Wilk test (p < 0.001), the Mardia skewness test (p < 0.001), and the Mardia Kurtosis test (p < 0.001). Finally, a display of residuals versus age revealed a heteroskedastic distribution implying that the variance of the error was not constant across ages (Fig. 2, panel d). Heteroskedasticity was confirmed with the Breusch–Pagan test (p < 0.001). OLS model is therefore insufficient to model longitudinal growth.

### Linear mixed-effect model without repeated measurements

The OLS model indicated that additional modeling components are necessary to account for individual-level clustering and residual autocorrelation. Linear mixed-effect models allow for non-independence and clustering by describing both between and within individual differences. We added random intercepts and slopes to the OLS model described above. Specifically, we added the parameters of the variance–covariance matrix to the fitted model (Table 2). Our model assumes that the variance–covariance of the random effects is unstructured.

At first, we only included random intercepts (b_0i_). The model assumes that the random intercepts are normally distributed with mean 0 and variance g_11_. We found that this parameter was statistically significant with a variance (g_11_) of 6.31 (95 % CI 5.21–7.62; p < 0.001). This result suggests that the growth of individual children diverge from the population prediction by a shift in the intercept; in other words, individual growth is shifted up or down but parallel to the population prediction.

Then, in a second step, we included both random intercepts and random slopes (b_1i_). The variance of the intercept (g_11_) was 3.60 (95 % CI 2.97–4.36; p < 0.001), the range is -5.27 cm to 3.57 cm and the mean is 0.0056 cm. In addition, the variance of the slope (g_22_) was 0.0091 cm/month (95 % CI 0.0075–0.011; p < 0.001), the range is −0.27 cm/month to 0.24 cm/month and the mean is 0.00077 cm/month (Table 3). Following the same reasoning as for random intercepts, the random slopes represent the individual variability in growth velocity around the population prediction: some individual children grow faster or slower than the population. A statistically significant variance, close to 0, as in this case, means that the shift of the individual growth velocity from the population is statistically supported but it is small. Therefore, the data exhibit less heterogeneity in the random slope than in the random intercept, indicating that the main source of difference between trajectories is encapsulated in their birth height. The story could be different in other growth cohorts or on other outcomes different than growth such as blood pressure.

**Table 3:** List of estimated parameters for each individual model

Parameter	Variable	Ordinary least squares	Random effects	Random effects and CAR(1)
$\beta_{0}$	Intercept	48.17 (47.43, 48.90)	48.07 (47.66, 48.49)	47.97 (47.56, 48.38)
$\beta_{1}$	$t_{ij}$	4.47 (3.39, 5.55)	4.53 (4.21, 4.84)	4.65 (4.33, 4.96)
$\beta_{2}$	$t_{ij}^{2}$	−0.28 (−0.75, 0.18)	−0.29 (−0.42, −0.15)	−0.32 (−0.47, −0.18)
$\beta_{3}$	$t_{ij}^{3}$	0.0015 (−0.061, 0.058)	−0.0014 (−0.019, 0.016)	0.0021 (−0.017, 0.021)
$\beta_{4}$	$\left( {t_{ij} - 3} \right)_{ + }^{3}$	0.026 (−0.044, 0.096)	0.026 (0.0062, 0.047)	0.023 (−0.00037, 0.046)
$\beta_{5}$	$\left( {t_{ij} - 6} \right)_{ + }^{3}$	−0.021 (−0.035, −0.0069)	−0.022 (−0.026, −0.018)	−0.022 (−0.028, −0.016)
$\beta_{6}$	$\left( {t_{ij} - 12} \right)_{ + }^{3}$	−0.0021 (−0.0063, 0.0020)	−0.0025 (−0.0037, −0.0013)	−0.0030 (−0.0050, −0.00097)
$\beta_{7}$	$\left( {t_{ij} - 18} \right)_{ + }^{3}$	−0.0018 (−0.0050, 0.0013)	−0.0011 (−0.0020, −0.00016)	−0.00056 (−0.0020, 0.00090)
$\beta_{8}$	$\left( {t_{ij} - 24} \right)_{ + }^{3}$	0.00092 (−0.00085, 0.0027)	0.00047 (−0.000038, 0.00098)	0.00032 (−0.00050, 0.0011)
$\beta_{9}$	$\left( {t_{ij} - 40} \right)_{ + }^{3}$	−0.019 (−0.040, 0.0030)	0.00019 (−0.0062, 0.0066)	−0.0029 (−0.011, 0.0048)
$\beta_{10}$	$I\left( {t_{ij} > 24} \right)$	−1.20 (−1.63, −0.77)	−0.91 (−1.03, −0.78)	−0.72 (−0.84, −0.60)
$\beta_{11}$	$I\left( {male} \right)$	1.64 (1.54, 1.74)	1.61 (1.11, 2.12)	1.51 (0.99, 2.02)
$g_{11}$	${\text{var}}\left( {\text{Intercept}} \right)$	–	3.60 (2.97, 4.36)	3.37 (2.73, 4.16)
$g_{22}$	${\text{var}}\left( {t_{ij} } \right)$	–	0.0091 (0.0075, 0.011)	0.0067 (0.0054, 0.0084)
$g_{12}$	${\text{var}}\left( {{\text{Intercept}}, t_{ij} } \right)$	–	0.019 (−0.0059, 0.044)	0.036 (0.013, 0.059)
$\rho$	Correlation	–	–	0.66 (0.64, 0.68)
$\sigma^{2}$	Unexplained variance	7.34 (−7.05, 21.76)	0.60 (0.59, 0.62)	0.81 (0.75, 0.87)

In our data, it was suitable to assume that the random effects follow an unstructured matrix because the random intercepts and random slopes have different variance estimates; this flexibility confers individuality to each child’s pattern of growth. The covariance (*g*_*12*_) describes how the random intercept varies with the random slope. The covariance for this data set was 0.019 (95 % CI −0.0059 to 0.044; p < 0.001; Table 3). A positive covariance, as what is shown from these data, suggested that children with greater individual intercept tend to have a greater rate of growth.

The unexplained or residual variance in this model is 0.60 (95 % CI 0.59–0.62). The introduction of random effects reduced the residual variance by an order of magnitude from 7.34 to 0.60, implying that individual effects explain a considerable portion of the outcome variation.

**Fig. 3:**
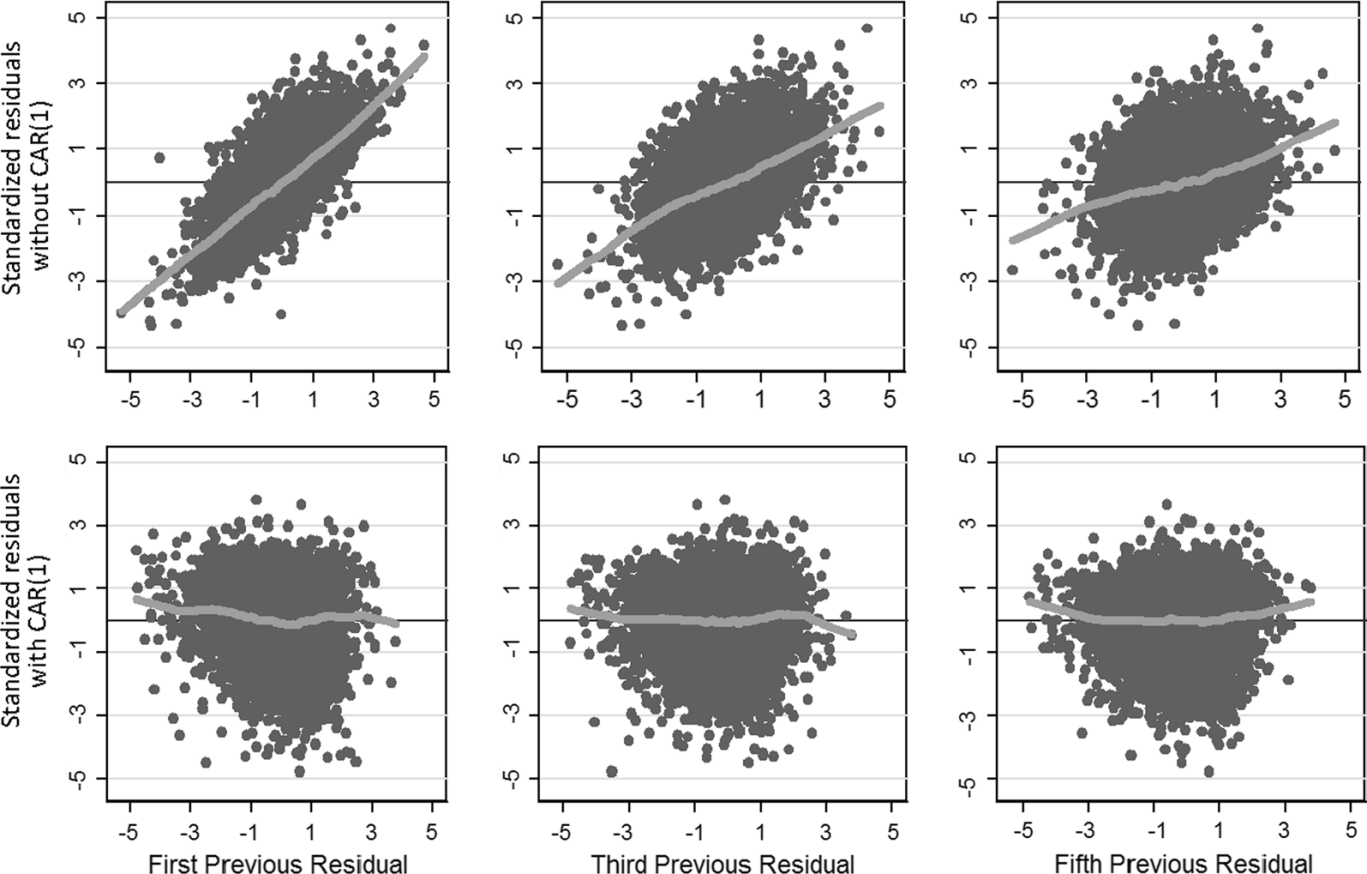
This graph plots the standardized residuals versus the first, third and fifth previous residual. The first lane (*top row*) are residuals form a fit using a linear mixed model without CAR(1) and the second with the inclusion of CAR(1). Notice how the autocorrelation is successfully treated with this approach

To evaluate the fit of our model, we visualized standardized residuals with age (Fig. 2, panel e). The linear mixed-effect model eliminated heteroskedasticity of residuals. The mixed model assumes errors are normal and conditionally independently distributed with mean zero and common variance. However, the estimated residuals did not appear randomly distributed; instead, they show symmetry in wider or narrower areas across the horizontal axis. Additionally, the variogram of the residuals still displays a degree of autocorrelation (see Fig. 2, panel b). The correlation in the residuals was confirmed by plotting the residual versus the residual of the previous observation within a child (Fig. 3). Taken together, a mixed model was able to address the non-independence and clustering that OLS could not, and in doing so account for a greater proportion of unexplained variance and reduce the heteroskedasticity. However, it still had limitations in addressing the serial correlation from repeated measures in growth data.

### Linear mixed-effect model with CAR (1) error for repeated measurements

From the exploratory data analysis, we noted that serial height measurements within children were auto-correlated. We used a CAR(1) error to capture serial correlation in our statistical model. This structure assumes that errors are correlated and the degree of correlation is greater in those closer with age than in those further apart. The third model adds a new parameter of correlation ρ = 0.66 (95 % CI 0.64–0.68; p < 0.001). To evaluate the fit of this model, we visualized residuals with age (Fig. 2, panel f). The autocorrelation was further reduced as evidenced by the variogram of the residuals and by the lack of relationship in scatter plots of residuals versus the previous observation’s residuals (Fig. 3). Both the random intercept and the random slope were statistically significant and followed the same growth patterns as before (Table 3). The covariance was 0.036 (95 % CI 0.013–0.059; p < 0.001). The unexplained variance was 0.81, it remained low and was similar to the unexplained variance of the linear mixed-effect model. Since there can be sex differences in child growth, we also evaluated the contribution of sex as a covariate. If all the variables remained constant, boys were on average 1.5 cm (95 % CI 0.99–2.01) taller than girls during the follow up period (Table 3).

### Cubic versus linear regression splines

Cubic regression splines were superior to linear regression splines in both estimation and prediction at each modeling step, and even when varying the number and position of knots. In Table 4, we report the values of AIC and BIC for OLS, LME, and LME with CAR(1) models even when the number of knots and their positions are varied. All results indicate that mixed effects models with cubic splines by far outperform the other models considered and that cubic splines outperform linear splines for every level of model complexity. AIC and BIC results show that cubic splines outperform linear splines even when cubic splines use three knots and linear splines use five knots. This is probably because the growth curvature is better captured by a cubic function than by multiple piecewise linear splines. Equally importantly, however, is the important reduction in AIC and BIC noted when a CAR(1) error structure was incorporated into the regression model.

**Table 4:** Comparing the Akaike information criterion and Bayesian information criterion for linear and cubic spline models using OLS (fixed effects only), LME (random slope random intercept) and LME with CAR(1) errors

	Linear splines		Cubic splines	
	Akaike information criterion			
	3 knots (3, 10, 29)	5 knots (3, 6, 18, 24)	3 knots (3, 10, 29)	5 knots (3, 6, 18, 24, 40)
Ordinary least squares	52,495.44	52,472.74	52,399.32	52,397.75
Random effects	28,608.28	28,345.14	27,560.38	27,541.87
Random effects and CAR(1)	19,719.72	19,495.80	19,222.76	19,235.37
Bayesian information criterion				
Ordinary least squares	52,553.77	52,545.24	52,472.23	52,485.24
Random effects	28,688.47	28,439.91	27,655.15	27,651.22
Random effects and CAR(1)	19,807.21	19,597.86	19,329.66	19,352.00

**Fig. 4:**
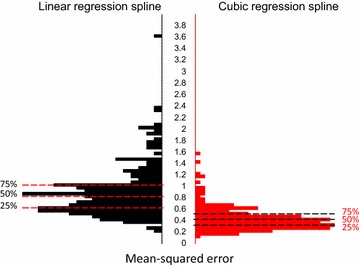
Subject-specific distributions of the square root MSEs for the entire growth curve for linear splines (*black*) versus cubic splines (*red*). Dashed lines correspond to the estimated median, 25 %, and 75 % percentiles of the subject-specific MSE distribution. Cubic regression splines outperformed piecewise linear splines: the median square root subject-specific MSE for linear regression splines was 0.65 vs. 0.51 for cubic regression splines. The Kolmogorov–Smirnov test indicates that the two distributions are significantly different (D = 0.19, p = 0.001)

Cubic regression splines models were also better at both estimation and prediction than were linear regression splines. Using three knots (at 3, 10, and 29 months) we obtained a median subject-specific estimation MSEs of 0.65 for linear regression splines and 0.51 for cubic regression splines (Fig. 4). A Kolmogorov–Smirnov test comparing the MSE distributions indicates that the two MSE distributions were different (D = 0.186, p = 0.001). Differences in the median subject-specific estimation MSE were similar even when the number of knots were increased, or their positions were varied (data not shown). Specifically, the out-of-sample prediction MSE was 0.86 for linear regression splines and 0.80 for cubic regression splines. These findings suggest that objective of inference in child growth problems should be primarily the curve and only secondarily the coefficients.

### Longitudinal models for height velocity and acceleration

With an appropriate model for height, the model for height velocity using a cubic regression spline is simply obtained from its first derivative (Table 1). Similarly, growth acceleration can be obtained by taking the derivative of the height velocity (formulas not shown). As a result of the derivation, the height velocity model loses the indicator variable of a difference between length and height. Also, sex is not included because our model assumes there is no interaction between sex and age. In this height velocity model, the random slope component is represented by ${\hat{\text{b}}}_{{1{\text{i}}}}$.

**Fig. 5:**
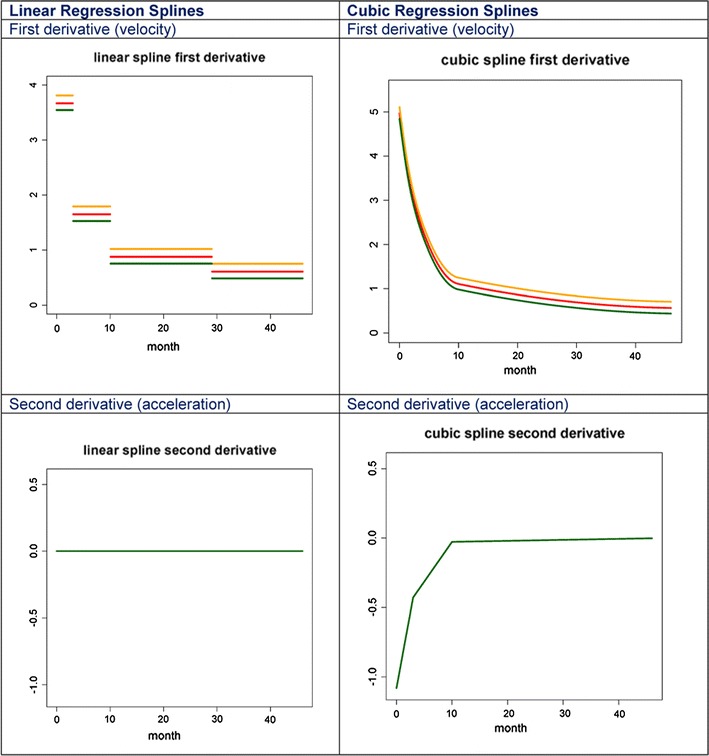
Estimated growth velocity (*top panels*) and acceleration (*bottom panels*) for three subjects in the study using linear (*left panels*) and cubic (*right panels*) splines with three knots (at 3, 10, and 29 months). A different number of knots and knot locations would result in slightly different plots, but with the same qualitative interpretation

An interesting characteristic of the height velocity and acceleration curves from a cubic regression model is that they are continuous and make biological sense. In contrast, a linear regression spline would assume that growth velocity is piecewise constant within knots but discontinuous across knots. Moreover, growth acceleration curve is zero. Both these assumptions of linear spline approaches contradict scientific knowledge about growth trajectories and the data in our application. For example, in Fig. 5 we estimated growth velocity (top panels) and acceleration (bottom panels) for three subjects in the study using linear (left panels) and cubic (right panels) splines with three knots (at 3, 10, and 29 months.) A different number of knots and knot locations would result in slightly different plots, but with the same qualitative interpretation. The linear spline plot assumes that growth velocity is piecewise constant between the knots, an assumption that once highlighted is very difficult to accept from a scientific perspective. In contrast, the cubic spline estimate of the velocity curve is much more aligned with the current knowledge of human growth with the velocity curve being continuous and smooth. Even more striking, the acceleration curve estimated using linear splines is always zero. In contrast, cubic regression splines estimate a negative acceleration, which is especially large in absolute terms in the first part of the curve. This corresponds to the obvious patterns we observe in growth curves: they have a slightly concave shape, with concavity much more pronounced immediately after birth (indicating deceleration of growth). Interestingly, the cubic spline estimator gets much closer to zero after month 10, but continues to be negative, which may indicate continuous concavity of the function, though much subtler.

### Interpretation of the final model

**Fig. 6:**
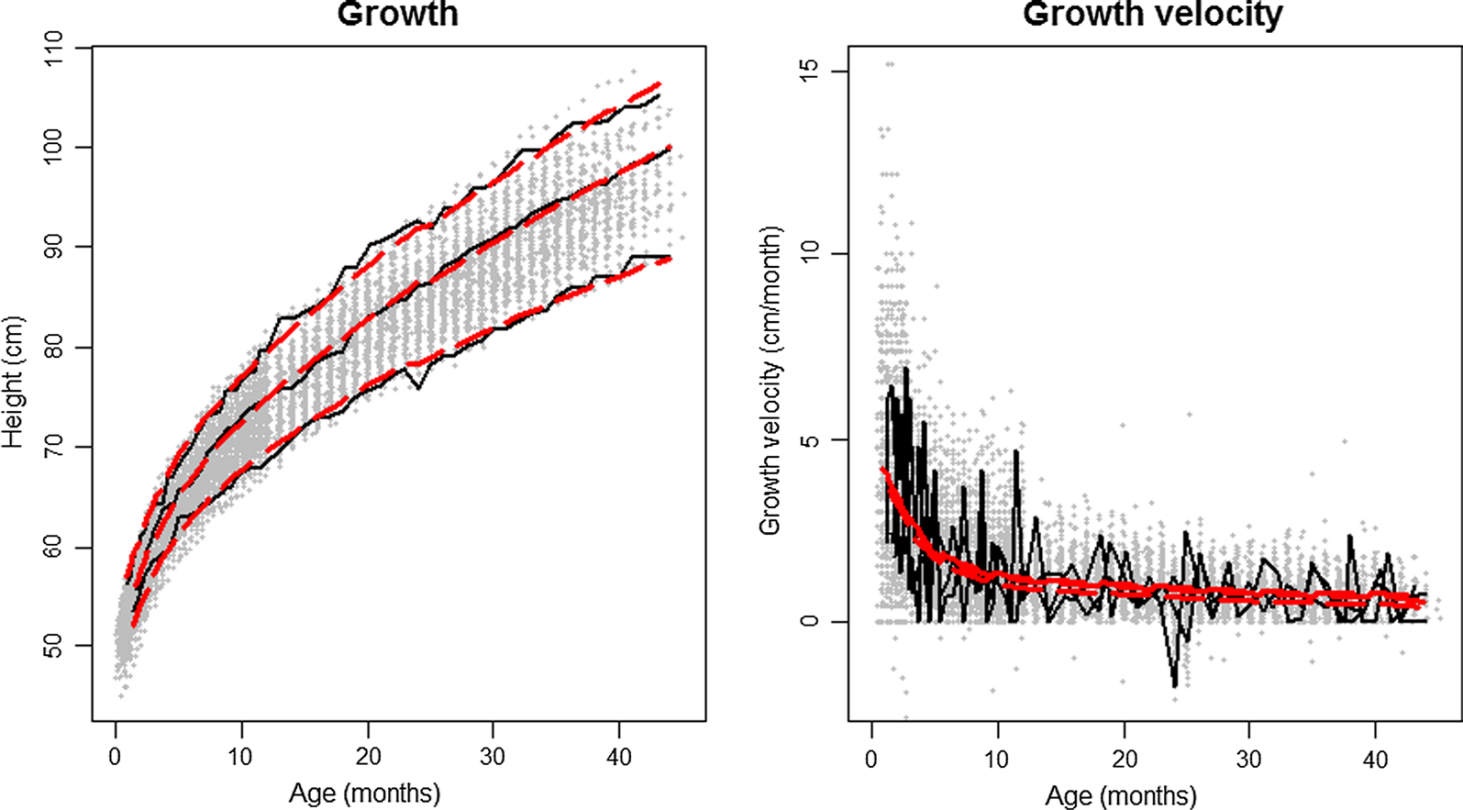
Observed and predicted growth for 3 individual children at different percentiles. In *black* are the observed growth curves and in *red* the predicted growth curves. The same children were plotted in both *graphs*. Notice children have a different growth pattern but similar growth velocity

By displaying the individual growth of three children at three different percentiles and their predicted values, we can see how our model effectively reflects individual and population growth patterns (Fig. 6). Differences between children appear to be largely from shifts in intercepts, with minimal differences in growth rates. This is further supported in the height velocity model in which the three individuals have almost the same rate of growth, only differing by a minimal vertical shift (Fig. 6). Therefore, the last model was able to effectively predict both population growth and differences between and within individuals in a population of Peruvian children from a peri-urban shanty town.

## Discussion

The final statistical model for the prediction of height was obtained by applying a linear mixed-effect model with random intercepts and slopes, and a first-order continuous autocorrelation structure for the error term. To account for the curvature of growth with age, we used cubic regression splines. To describe this modeling process, we build longitudinal growth data using a stepwise process. At each step, we examined standardized residuals with age, the sample variogram of the residuals and pairwise scatterplots of residuals at different time points to assess goodness-of-fit. We also compared estimation and prediction of cubic regression spline models vis-à-vis linear regression splines, and found that the former outperform the latter. This suggests that inference of child growth problems should focus on differences in growth curves based on the regression parameters rather than on direct interpretation of the regression parameters themselves.

While a sizeable literature on modeling child growth data already exists [1–11], it should be recognized that estimation and, more importantly, prediction of subject-specific growth trajectories continues to be a problem under active methodological development and much attention needs to be given to the various choices and refinements to address the wide variety of applications. Our modeling framework adds to the literature in at least three directions. First, we are interested in fitting and predicting both at the population and subject-specific level, whereas previous related papers focus exclusively on population-level parameters [6–11]. In large studies with more than 100 children, estimating the population level parameters is relatively easy and straightforward and there is not a lot of need for modeling the subject-specific deviations. However, subject-specific estimation and predictions is more complicated and requires a higher level of technical detail. Second, cubic regression splines provide a better fit than linear splines to non-linear growth curves when the number of knots is small, as proposed by us and others. This happens because cubic regression splines capture better the non-linear trajectories between knots and are especially well-suited for modeling child growth immediately after birth when both acceleration and velocity are the greatest. Second, cubic regression splines assume that the velocity of growth in children is continuous and smooth whereas linear splines assume that velocity is constant in a small number of intervals, an assumption that is biologically difficult to support. Moreover, growth acceleration is a piecewise linear and continuous function when data are modeled as cubic splines, whereas a linear spline approach assumes that growth acceleration is exactly zero. Our results indicate that, for this growth data set, cubic splines outperforms linear splines when the same number and location of knots is used, and these findings are consistent with previously published work [26, 27]. In general, we recommend conducting application-specific simulations to assess which model performs better. Third, modeling residuals as a continuous auto-regressive process is a fundamentally important feature of unbalanced data that needs to be taken carefully into account.

Linear mixed-effect models are an advantageous and appropriate statistical method for longitudinal growth in children under 5 years of age. Linear mixed models not only provide information about the population prediction of growth but also give insight on individual patterns of growth through the random component. Our analysis also points to the importance of adequately accounting for autocorrelation of repeated measurements. Inclusion of cubic regression splines when compared to linear splines to model the curvature of growth is supported by our data because more measurements were taken when growth was faster. Also, linear mixed models allow for the analysis of unbalanced data with ease, e.g. subjects with a different number of observations and observations measured at different points in time. From a methodological and logistical perspective, this is an advantage because sometimes it is hard to ensure that subjects do not miss any visits and that they are measured at the exact scheduled time. Linear mixed-effect models can account for some bias because subjects with complete follow up time might differ from subjects lost during the follow up period [28]. In addition, linear mixed-effect models support several variance–covariance matrices and structure of the residuals, allowing flexibility for various types of data with different levels of clustering, the inclusion of time-varying and time-invariant covariates and an efficient method to account for repeated measurements [17, 18].

A limitation of linear mixed-effect model approach presented in this paper is that not all the statistical software packages support the generation of variograms for longitudinal data, and may therefore require programming. Our analysis has some additional shortcomings. First, our inferences are based on the analysis of a single dataset. Second, the population under study consisted of a rather homogenous group of children with a high number of height measurements in early childhood. We acknowledge that there may be differences in statistical modeling approaches in study samples that exhibit more heterogeneity and have fewer measurements during early childhood. Third, our analytical approach requires the assumption that growth data are missing at random, in which case mixed effects models are very robust. If outcome data were not missing at random (e.g. the child was sick for a long period of time), then this can lead to larger prediction errors due to subject-specific biases. Finally, we did not include an interaction between sex and age to simplify our illustration of the statistical principles of our analysis; however, this model may not be realistic for human growth or perhaps for other longitudinal outcomes.

In conclusion, we present a stepwise approach to developing longitudinal growth models using linear mixed-effect models that account for random effects and serial autocorrelation with cubic regression splines to capture the non-linear relationship between age and growth in height. As compared to other approaches, this modeling approach is simpler, direct and does not require multiple steps of transformation, analyzing age intervals separately or estimating LMS parameters. The growth velocity model obtained from the derivative eliminates measurement error directly from the growth without the need of assuming negative increments as no growth. Moreover, models based on cubic regression splines outperform those based on linear regression splines, suggesting that inferences should be based on differences in growth curves based on parameters and not on the interpretation of the parameters themselves. Therefore, researchers who seek to model longitudinal child growth in their investigations would benefit from using these steps for mixed modeling in future analyses.

## Additional file

10.1186/s12982-015-0038-3 Examples of statistical code in R.

## Abbreviations

CAR(1): continuous autoregression of order 1
OLS: ordinary least squares
TPS: truncated polynomial spline
