# Supplementary material for: Modelling subject-specific childhood growth using linear mixed-effect models with cubic regression splines
Source: Emerg Themes Epidemiol. 2016 Jan 7;13:1. doi: 10.1186/s12982-015-0038-3 (PMC4705630; doi:10.1186/s12982-015-0038-3)

Child_growth_splines

Ciprian M Crainiceanu

Tuesday, July 21, 2015

This document provides the basic R code for building linear mixed effects models of various complexities for child growth data. We are focusing on models of the type

$$Y_i(t_{ij}) = X_i\gamma + \sum_{l=1}^{p}(\beta_l + \beta_{il})t_{ij}^l + \sum_{k=1}^{K} b_k(t_{ij} - \kappa_k)_+^p + \epsilon_{ij},$$

where X_i are covariates (e.g. sex), $(\beta_{i1}, \ldots, \beta_{ip})^t$ is a p-variate normal vector of mean zero random effects with a $p \times p$ unstructured covariance matrix Σ, $\kappa_1, \ldots, \kappa_K$ are the knots used for the splines, and p is the degree of the spline. In our context we use $p = 1$ for linear splines and $p = 3$ for cubic splines as well as $K = 3$ ($\kappa_1 = 3$, $\kappa_2 = 10$, $\kappa_3 = 29$) as used in the paper by Howe et al., 2013 and $K = 5$ ($\kappa_1 = 3$, $\kappa_2 = 6$, $\kappa_3 = 18$, $\kappa_4 = 24$, and $\kappa_5 = 40$). Note that the first derivative (growth velocity) can be estimated from the model as

$$p\sum_{l=1}^{p}(\beta_l + \beta_{il})t_{ij}^{l-1} + p\sum_{k=1}^{K} b_k(t_{ij} - \kappa_k)_+^{p-1},$$

which is a piece-wise constant function if $p = 1$ (linear spline) and is a continuous smooth function for $p = 3$ (cubic spline).

We start by setting up the data and labels in R.

Set up the working directory (we chose a working directory that contains the dataset)

```
setwd("D:/collaborators/OTHERS/Andrada_Ivanescu/lme_resubmission/data_and_software")
```

Upload the necessary libraries (lme4 and nlme are essential)

```
## necessary libraries:
library(nlme)
library(foreign)
library(ggplot2)
library(lme4)
```

```
## Loading required package: Matrix
##
## Attaching package: 'lme4'
##
## The following object is masked from 'package:nlme':
##
##     lmList
```

Read in the data, check the variable names, and attach the data. Data are contained in the file named "Complete database.dta"

```
file.dta = read.dta("Complete database.dta")
names(file.dta)
```

```
##  [1] "codigo"      "fechaentr"   "fechanac"    "height"      "edaddias"   
##  [6] "edadmes"     "gender"      "weight"      "BMI"         "waz"        
## [11] "haz"         "baz"         "whz"         "fwasting"    "fstunting"  
## [16] "funweight"   "fBMIag"      "heightbr"    "weightbr"    "px"         
## [21] "ma0fe1"      "ltxht"       "educmat"     "cgeocasa"    "tipocasa"   
## [26] "almagua"     "elimexcret"  "tierefrig"   "tiedvdvhs"   "tieminest"  
## [31] "tietelef"    "tiecompu"    "tiecarro"    "inmensfam"   "gastmagua"  
## [36] "gastmluz"    "gastmcom"    "lost"        "baseline"    "atierefrig" 
## [41] "atiedvdvhs"  "atieminest"  "atietelef"   "atiecompu"   "atiecarro"  
## [46] "assets"      "gastos"      "salmagua"    "selimexcret" "aguasani"   
## [51] "peso_rn"     "talla_rn"    "talla_ma"    "difheightbr" "Aheight"    
## [56] "Aweight"
```

```
attach(file.dta)
```

Define the unique ids (subjects). The function unique in R ignores the repetition of the same id and keeps only the unique ids. n stores the number of subjects

```
ui<-unique(file.dta[,"codigo"])
n=length(ui)
```

Transform the id codes stored in codigo into a number between 1 and n=215. id.num has a length of 10838, which is the total number of growth measurements for the 215 children (average of 50.4 observations per child)

```
file.dta$id.num<-as.numeric(as.factor(file.dta$codigo))
id.num<-as.numeric(as.factor(file.dta$codigo))
length(id.num)
```

```
## [1] 10838
```

Build the necessary ingredients for teh linear mixed effects model using a truncated cubic spline approach 5 knots at $\kappa_1 = 3$, $\kappa_2 = 6$, $\kappa_3 = 18$, $\kappa_4 = 24$, and $\kappa_5 = 40$. Start by buiding the linear, quadratic, and cubic monomials of age

```
t = edadmes
t2 = t^2
t3 = t^3
```

Build the truncated cubic splines

```
t3.3=(t-3)^3*I(t>3)
t3.6=(t-6)^3*I(t>6)
t3.18=(t-18)^3*I(t>18)
t3.24=(t-24)^3*I(t>24)
t3.40=(t-40)^3*I(t>40)
```

Fitting an OLS (no mixed effects) truncated cubic splines (this model does not allow for subject-specific random effects, $\beta_{il}.$). This model is not appropriate for subject-specific fitting, but can be used for estimation of population-level parameters. Below, the variable ma0fe1 is the sex variable that encodes male as 0 and female as 1.

```r
fit.lme.CUB.ols.5 = lm(height ~ factor(ma0fe1) + I(t>24) + t+ t2 + t3 +
                              t3.3  + t3.6 + t3.18+t3.24+t3.40)
```

Obtain and display AIC and BIC for this model

```r
AIC(fit.lme.CUB.ols.5)
```

```
## [1] 52397.75
```

```r
BIC(fit.lme.CUB.ols.5)
```

```
## [1] 52485.24
```

Fitting a linear mxed effects model where we allow for random intercept and random slopes (the quadratic and cubic terms do not have random effects) with truncated cubic splines

```r
fit.lme.CUB.5 = lme(height ~ factor(ma0fe1) + I(t>24) + t+ t2 + t3 +
                             t3.3  + t3.6 + t3.18+t3.24+t3.40,
                             random= ~1+t|id.num,method='REML')
```

Obtain and display AIC and BIC for this model

```r
AIC(fit.lme.CUB.5)
```

```
## [1] 27541.87
```

```r
BIC(fit.lme.CUB.5)
```

```
## [1] 27651.22
```

Fit the same model, but accounting for AR(1) correlation of residuals

```r
fit.lme.CUB.ar.5 = lme(height ~ factor(ma0fe1) + I(t>24) + t+ t2 + t3 +
                              t3.3 + t3.6 + t3.18 + t3.24 + t3.40,
                              random= ~1+t|id.num,
                              correlation=corCAR1(form=~t|id.num),method='REML')
```

Obtain and display AIC and BIC for this model

```r
AIC(fit.lme.CUB.ar.5)
```

```
## [1] 19235.37
```

```r
BIC(fit.lme.CUB.ar.5)
```

```
## [1] 19352
```

We would also like to plot the data for a couple of subjects together with their corresponding fits. We will focus on subject 1 and subject 150.

```
su=1
plot(t[id.num==su],height[id.num==su],xlab="Time (months)",ylab="Height (cms)",pch=20)
lines(t[id.num==su],fit.lme.CUB.5$fitted[id.num==su,2],col="blue",lwd=3)
```

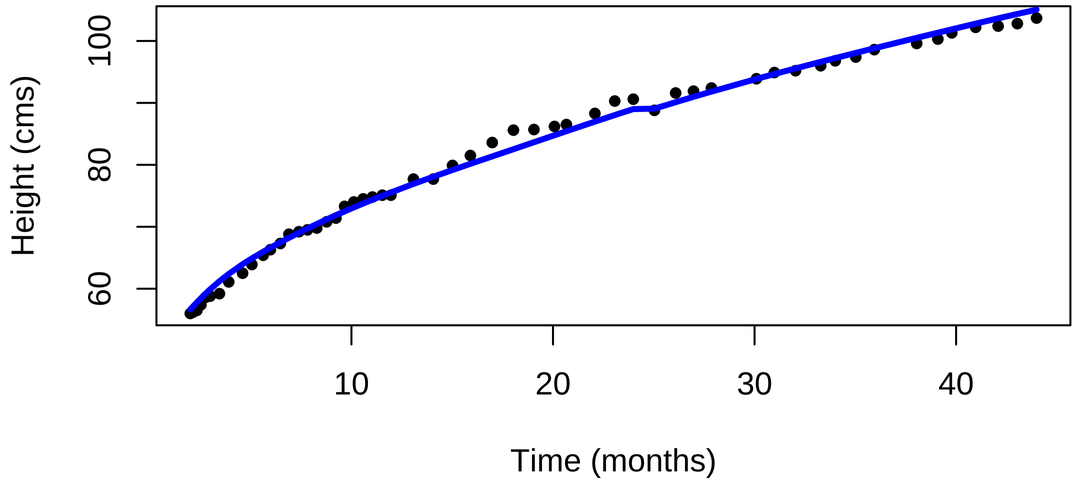

Do the same thing for subject 150

```
su=150
plot(t[id.num==su],height[id.num==su],xlab="Time (months)",ylab="Height (cms)",pch=20)
lines(t[id.num==su],fit.lme.CUB.5$fitted[id.num==su,2],col="blue",lwd=3)
```

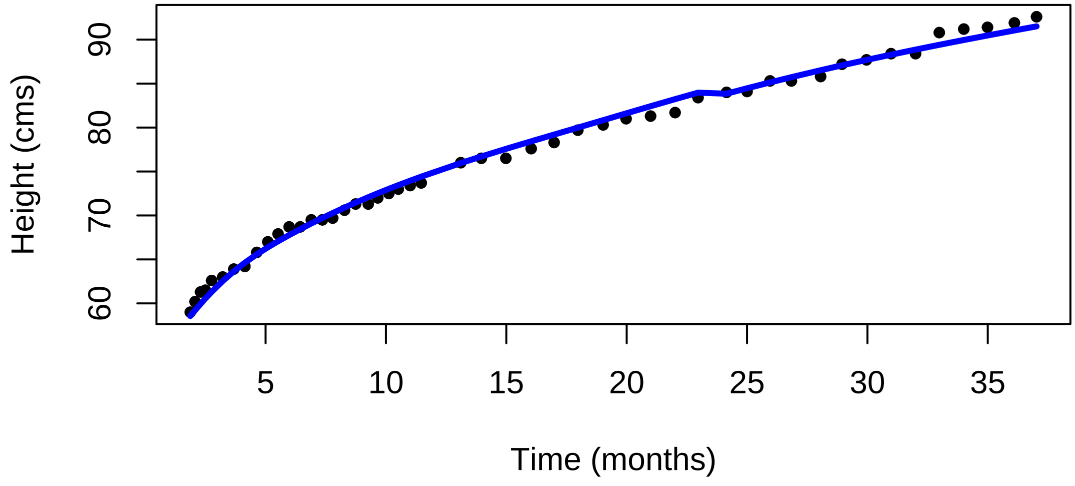

Supplement: Supplementary file 1 — 10.1186/s12982-015-0038-3 Examples of statistical code in R. [file 12982_2015_38_MOESM1_ESM.pdf]
